# Intramedullary osteosynthesis versus plate osteosynthesis in subtrochanteric fractures

**Published:** 2011-11-24

**Authors:** C Burnei, Gh Popescu, D Barbu, F Capraru

**Affiliations:** *,,Carol Davila’’ University of Medicine and Pharmacy, Bucharest, Department of Orthopedics, Clinical Emergency Hospital Bucharest, Romania; ** Department of Orthopedics, Clinical Emergency Hospital, Bucharest, Romania

**Keywords:** subtrochanteric fracture, intramedullary nailing, plate osteosynthesis

## Abstract

Due to an ever-aging population and a growing prevalence of osteoporosis and motor vehicle accidents, the number of subtrochanteric fractures is increasing worldwide.

The choice of the appropriate implant continues to be critical for fixation of unstable hip fractures. The subtrochanteric region has certain anatomical and biomechanical features that can make fractures in this region difficult to treat. The preferred type of device is a matter of debate. Increased understandings of biomechanical characteristics of the hip and improvement of the implant materials have reduced the incidence of complications. The surgeons choose between the two methods according to Seinsheimer's classification and also to their personal preferences. As a general principle, the open reduction and internal fixation were performed in stable fractures, and the closed reduction and internal fixation were performed in unstable fractures.

The advantages of intramedullary nailing consist in a small skin incision, lower operating times, preservation of fracture hematoma and the possibility of early weight bearing. The disadvantages consist in a difficult closed reduction due to important muscular forces, although the nail can be used as a reduction instrument, and higher implant cost.

In open reduction internal fixation techniques, the advantage is represented by anatomical reduction which, in our opinion, is not necessary. The disadvantages are represented by: higher operating time, demanding surgery, large devascularization, higher infection rates, late weight bearing, medial instability, refracture after plate removal and inesthetic approach.

## Introduction

Due to an ever-aging population and a growing prevalence of osteoporosis and motor vehicle accidents, the number of subtrochanteric fractures is increasing worldwide [**1**]. These fractures are associated with a mortality percentage of 5% up to 15% in elderly, functional disability, and loss of mobility and independence with a negative impact on the patient’s life and on healthcare costs [**1,2,3**]. Subtrochanteric fractures are relatively rare, accounting for 10 to 34% of all hip fractures [**4**]. They may be very difficult to fix, and the risk of failure has been high regardless of the fixation method. Methods to improve the medical care for these patients that allow early mobilization and a fast return to initial status are required [**5**]. A large variety of implants for the treatment of hip fractures is available to address these types of complications. However, if different implants are used for unstable fractures (comminuted, medial instability) there might be considerable differences in results [**2,6**]. The choice of the implant is particularly critical in unstable fractures [**7,8,9**]. This is reflected in the clinical situation in which the failure rate in unstable fractures is significantly increased compared to stable fractures. These complications in unstable fractures are largely related to the local mechanical situation at the fracture site and wrong implant selection. Fractures in the subtrochanteric region are difficult to treat because of their anatomical and biomechanical features [**1,9**]. Restoration of femoral length, rotation and correction of femoral head and neck angulations are of high importance [**5**]. This can be achieved only by an operative treatment. The orthopedic treatment is not accepted anymore due to a higher death rate and unsatisfactory results [**2,3,8,9**]. It is reserved only for the cases in which surgery is not an option.

## Materials and methods

From August 2009 to August 2011 we treated 68 patients with primary subtrochanteric fractures and 7 patients who needed a second intervention due to implant failure or other complication who underwent the initial surgery in other clinics. The mean age of the patients was 64 years (range: 29–87 years) and the sex distribution was 41 males and 27 females. In 63% cases the fracture was the result of a fall and in 37% of cases was caused by a motor vehicle accident. No pathological fracture, open fracture or polytrauma were included in this study.

**Table 1:** Age/sex and fracture type representation

Type of fracture	Patients	Male	Female
Stable subtrochanteric	23	13	10
Unstable subtrochanteric	31	19	12
Unstable subtrochanteric	12	9	3
Combination of injuries	2	0	2
Second intervention needed	7	5	2

Plain radiographs were taken on admission, including anterior-posterior (AP) pelvis. AP and lateral calibrated plain radiographs of the entire femur were also obtained to decide on implant length [**1,2,8,9**]. The primary assessment included categorization of the fractures according to Seinsheimer’s classification which, in our opinion, is also the most comprehensive one [**1,2,5,7**]. (**Table 2**)[**10**].

**Table 2:** Seinsheimer's Classification (modified in conformity to original)[10].

Seinsheimer's Classification of Subtrochanteric Fractures
Type I:	- nondisplaced fracture: < 2 mm of displacement of fracture fragments;
Type II: two part fractures:	- IIA: Two part transverse femoral fracture	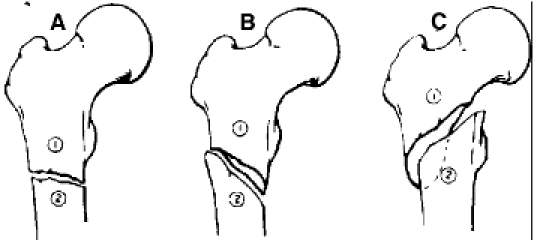
	- IIB: Two part spiral fracture with lesser trochanter attached to proximal fragment
	- IIC: Two part spiral fracture with lesser trochanter attached to distal fragment
Type III: three part fractures:	- IIIA -three part spiral fracture in which lesser trochanter is part of 3rd fragment which has an inferior spike of cortex of varying length -implant failures and non-unions are common	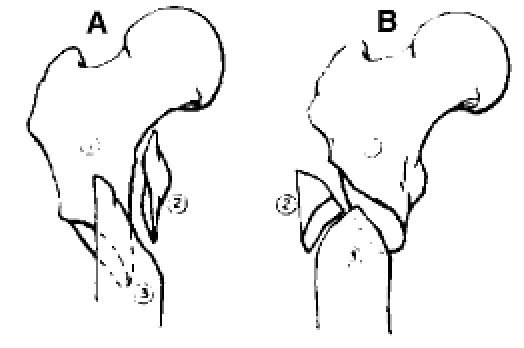
	- IIIB: -three part spiral fracture of proximal 1/3 of femur, with third part butterfly fragment
Type IV:	-comminuted fracture with 4 or more fragments -implant failures and non-unions are common	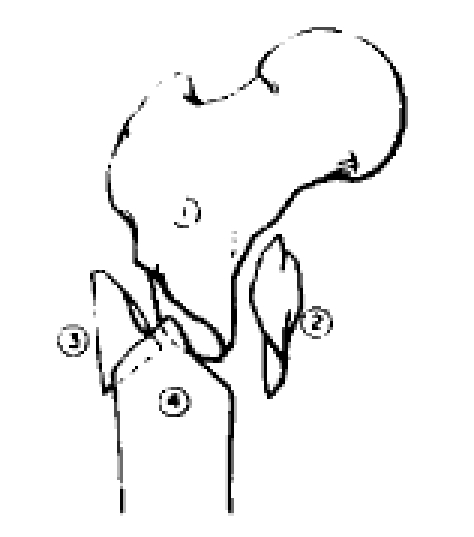
Type V:	-subtrochanteric intertrochanteric fractures; -this group includes any subtrochanteric fractures with proximal extension;	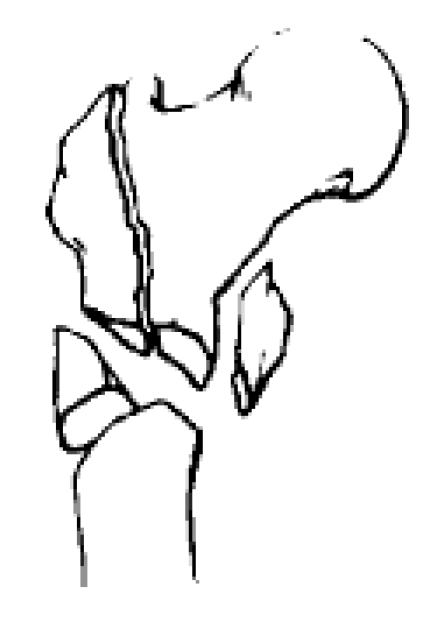

The functional status was recorded at the time of fracture and periodically at 3 weeks, 1 month and 3 months after surgical treatment using standardized forms with data that includes function, pain scale and mobilization with or without aids and also a radiographic status of bone healing. Function was expressed as a change compared to the situation at the time of fracture (same as before, worse, no weight bearing). Post surgical complication was also recorded in a separate form.

### Operative techniques.

The surgeons choose between the two methods according to Seinsheimer's classification and also to their personal preferences [**7**]. As a general principle the open reduction and internal fixation (O.R.I.F.) were performed in stable fractures, and the closed reduction and internal fixation were performed in unstable fractures. All surgeries were performed on an orthopedic surgery table under image intensifier. We used standard surgery techniques for gamma nail, proximal femoral nail (P.F.N.), dynamic hip screw (D.H.S.) and dynamic condylar screw (D.C.S.) [**1,2,7,8**]. The time elapsed from the patient’s presentation until the surgery was no longer than 48 hours. Thromboembolic and antibiotic prophylaxis were used in all cases. No bone grafts or bone substituent’s were used. The implants’ type and length were adapted to each case according to fracture pattern and extension according to A.O. principles and manufacturers specifications [**7**]. Standard Gamma nails were used in 22 patients, long Gamma nails in 9 cases, P.F.N. in 13 cases, DCS in 5 cases and DHS in 26 cases. In the 7 cases that required a reintervention we used a longer and thicker version of Gamma nail.

**Fig. 1:**
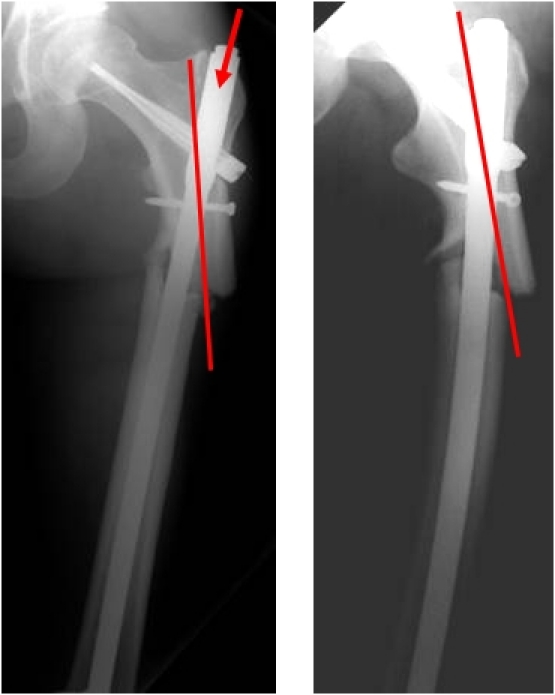
Varus deformation due to a wrong nail insertion point.

As a principle, we used a plate that permitted at least 3 screws over the distal part of the fracture and an additional antirotatory neck screw in fractures that extended to greater trochanter. Reaming was performed in all nailing cases. The patients were mobilized after an X–ray examination on the first postoperative day without weight bearing. Partial weight bearing was allowed according to each patient status between 2–4 weeks in O.R.I.F. and from the second day to 2 weeks in nailing cases. Full weight bearing was normally allowed 6–12 weeks after surgery based on the radiological status.

## Results

Seinsheimer’s fracture type IIIA was the most common fracture pattern. The average operating time for nailing group was 78 minutes, the hospitalization time approximately 6 days. The O.R.I.F. group shows an average operating time of 145 minutes and hospitalization of 9 days.

The differences between the groups were significant in surgery demanding and hospitalization cost disfavoring the plating group, although the implant cost are up to 45% higher in nailing favor.

Difficulties in reduction were slightly less common with plating than with nailing. In nailing group, difficulties in reduction and extension of operation were most frequent in type IIC fractures.

Supplementary fixation (cerclage wire) was required in 5 cases in the Gamma nail group and in 2 cases in the DHS group (antirotator screw).(**Fig. 2**)

**Figure:**
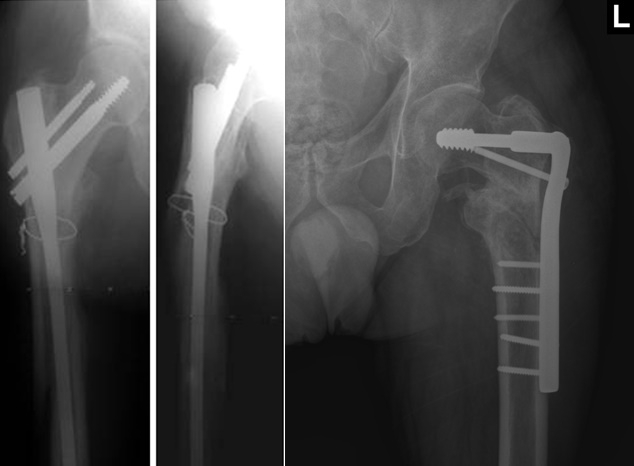
**Fig. 2**Additional reduction using cerclage wire (gamma nail) and antirotator screw (D.C.S.)

**Table 3:** Main complications

	Nailing group/nr.cases	Plating group/nr. cases	Shaft fracture intra or postoperative		Device failure and/or insufficient reduction obtained		Superficial infection( no bone infection encountered)		Reintervention due to complication	
Seinsheimer			Nailing	Plating	Nailing	Plating	Nailing	Plating	Nailing	Plating
I	0	0	0	0	0	0	0	0	0	0
II A	2	14	0	2	0	1	0	3	0	3
II B	3	9	0	0	0	0	0	1	0	0
II C	6	2	1	0	0	0	0	0	1	0
III A	13	3	1	0	1	0	0	0	1	0
III B	4	0	0	0	0	0	0	0	0	0
IV	6	2	0	1	0	1	0	1	0	2
V	3	1	0	0	1	0	0	0	1	0
TOTAL	37	31	2	3	2	2	0	5	3	5
Percentage	54.5%	45.5%	2.9%	4.4%	2.9%	2.9%	0.0%	7.3%	4.4%	7.3%

Postoperative complications *were more common in the plating group than in the nail group [**11,14,15**]* and they are depicted in **Table 3**.

In our study, 73% of the patients in the nail group and 68% in the plating group regained their initially walking ability, the other patients can also walk, but they accuse moderate pain and leg instability.

**Fig. 3:**
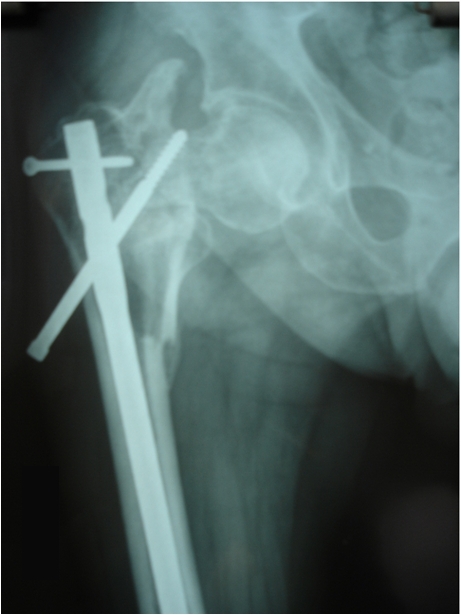
Implant failure due to insufficient stability in femoral neck and head.

The implant failure rate was similar in both groups (2.9%) and was mainly due to a bad assessment of fracture type, improper closed reduction, short plates, wrong entry point of the nail and early unsupervised mobilization.

**Fig. 4:**
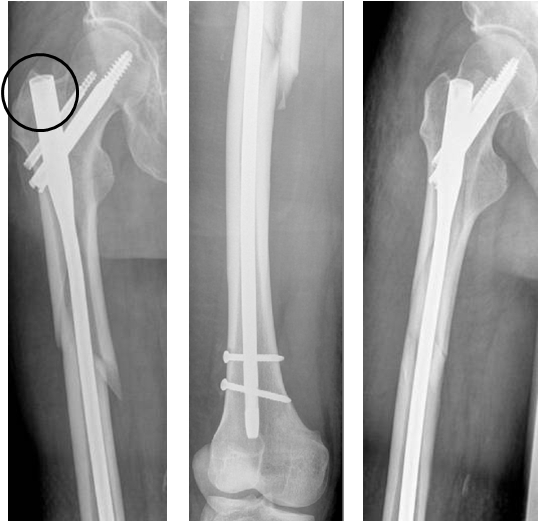
Showing correct entry point, correct implant length and that anatomical reduction is not necessary for a good outcome.

The need of surgical reintervention was significantly higher in plating group (almost double).

Luckily we did not encounter infection in the nailing group. In the plating group the rate of infection was 7.3% due to large exposure and longer surgery times as compared to closed reduction technique. They mainly consist in superficial tissue infection and were treated accordingly.

**Fig. 5:**
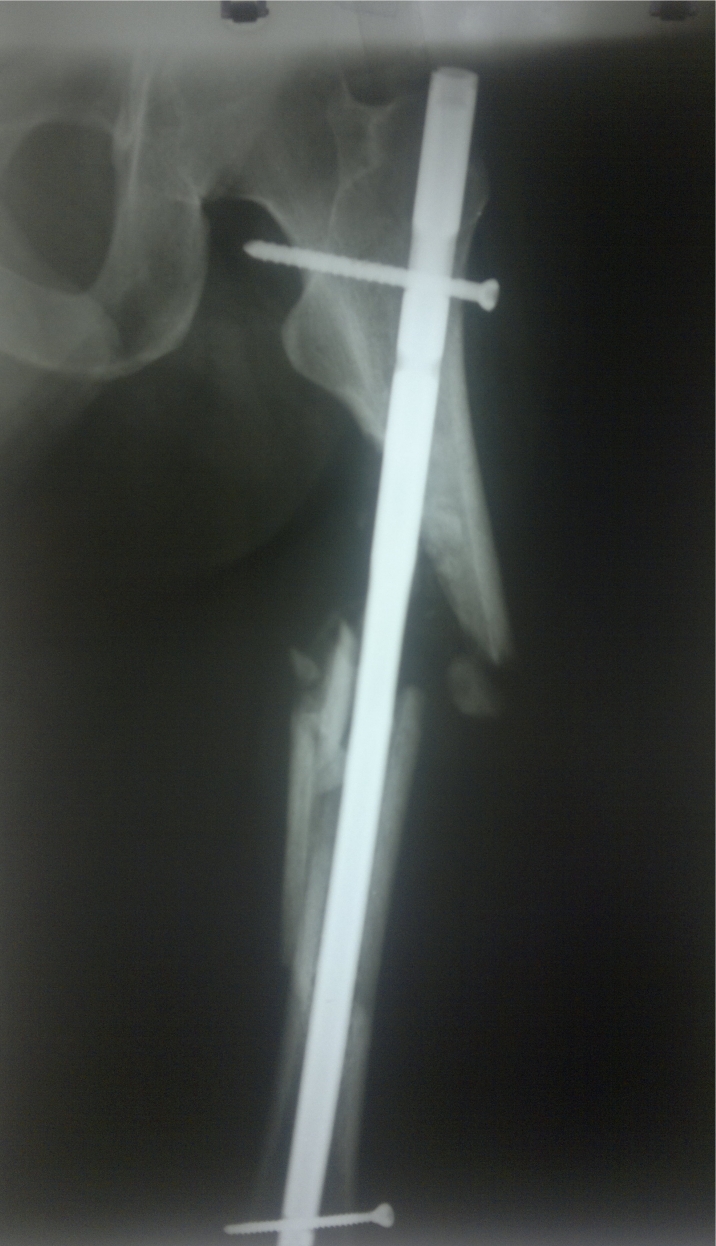
Incorrect insertion point and improper proximal looking leaded to important varus deformity.

We also included in this study 7 patients who underwent a primary failed intervention in other clinics. 5 of them presented at approximately 6 months after first intervention showing a nonunion varus deformity on P.F.N. osteosynthesis due to a wrong entry point at the trochanter level (**Fig. 5**).

**Fig. 6:**
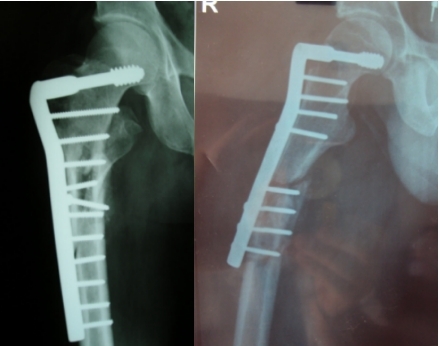
Secondary fracture due to insufficient number of distal screw and medial comminution.

One of the cases presented a short D.H.S. and a postoperative fracture at the level of the distal screw (**Fig. 6**).

The last case presented a D.H.S. ,,cut out’’ after a medial unstable fracture and a early weight bearing for this type of fracture. In all this cases we performed a long gamma nail osteosynthesis and distal locking. The operating time was significantly higher (between 4–6 hours), blood loose was important doe to the scar tissue and extensive reaming.

## Discussion

**Fig. 7:**
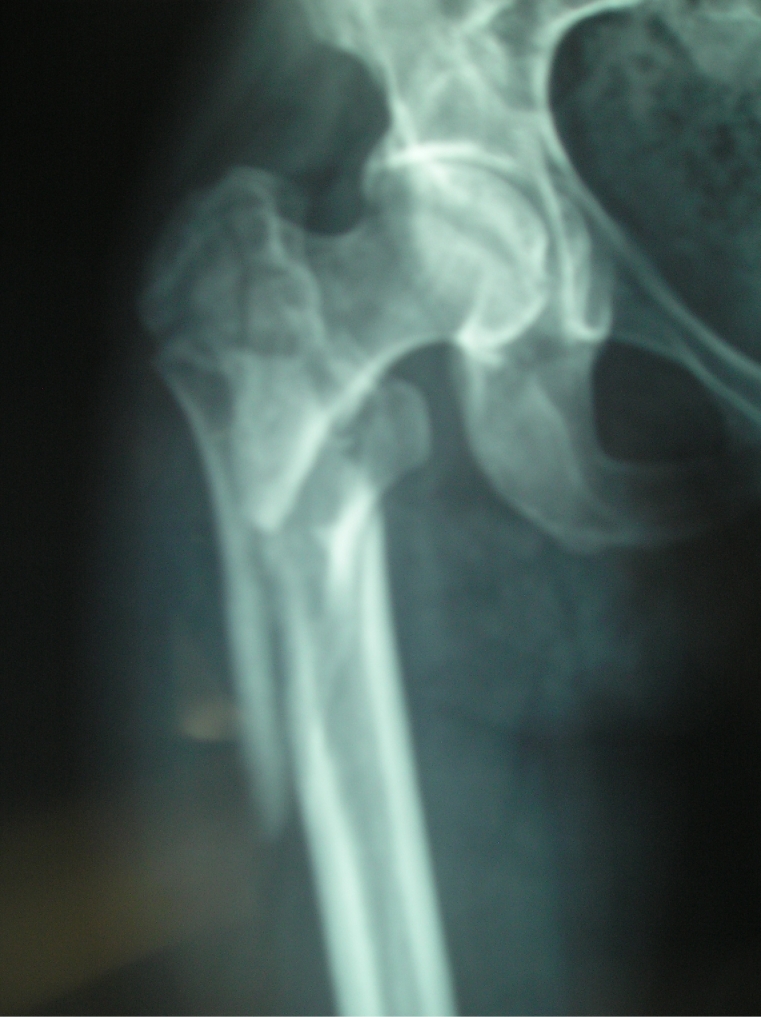
Fracture that extend to greater trochanter and to diaphysis.

The classification of subtrochanteric fractures is very difficult; [**3,4,5**] the actual borderline between trochanteric, subtrochanteric and dia–meta–epiphyseal fractures is unclear (**Fig. 7**).

In the evaluation of the results, we used Seinsheimer’s classification [**10**], which was applied in the majority of studies on subtrochanteric fractures and which, in our opinion, also proved the most practicable system [**1,2,3,4,5,7,8**].

The advantages of intramedullary nailing consist in [**11,14,15**]: small skin incision, lower operating times, fracture hematoma is preserved and early weight bearing is possible. The disadvantages consist in a difficult closed reduction due to important muscular forces although the nail can be used as a reduction instrument and a higher implant cost [**1,7**].

**Fig. 8:**
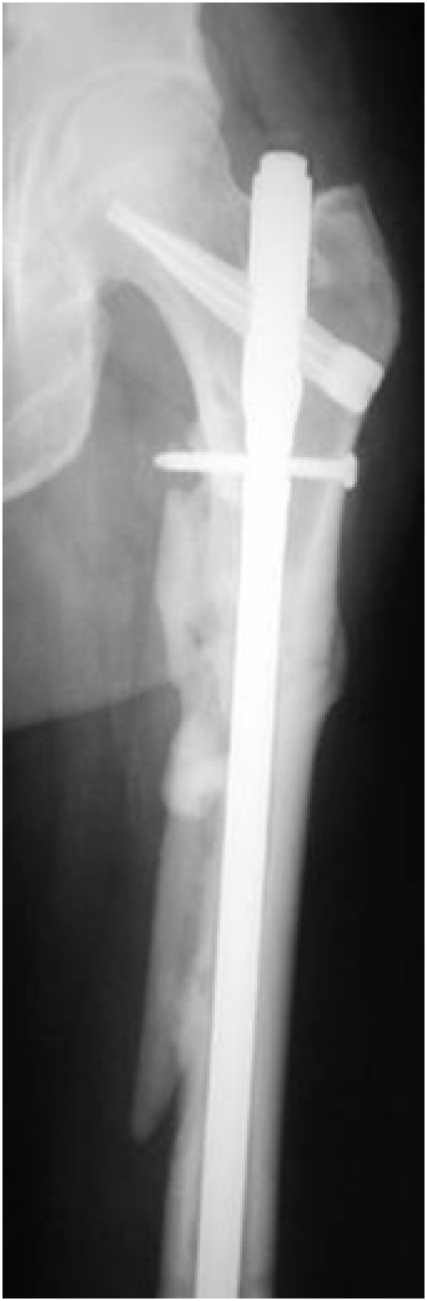
No anatomical reduction is necessary to obtain a proper healing

In open reduction internal fixation techniques, the advantage is represented by anatomical reduction which in our opinion in not necessary. The disadvantages are represented by: higher operating time, demanding surgery, large devascularization, higher infection rates, late weight bearing, medial instability, refracture after plate removal and inesthetic approach [**2,6,12,13**]. It was concluded that detailed fracture classification, restoration of length, rotation and alignment of fragments, is essential (anatomical reduction is not necessary) (**Fig. 8**).

The implant configuration and placement must be done according to the fracture type and weight bearing should be adapted accordingly to fracture type, implant type and bone quality.

We recommend that, despite the perioperative problems associated with nailing, this technique is preferable to plate fixation especially for specific fracture types with medial cortical comminution.
